# Elastodontic Devices in Orthodontics: An In-Vitro Study on Mechanical Deformation under Loading

**DOI:** 10.3390/bioengineering9070282

**Published:** 2022-06-28

**Authors:** Vincenzo Quinzi, Gianni Gallusi, Elisabetta Carli, Francesca Pepe, Elena Rastelli, Simona Tecco

**Affiliations:** 1 Department of Life, Health and Environmental Science, University of L’Aquila, 67100 L’Aquila, Italy; vincenzo.quinzi@univaq.it (V.Q.); gianni.gallusi@gmail.com (G.G.); Francesca.pepe@student.univaq.it (F.P.); elena.rastelli@student.univaq.it (E.R.); 2 Department of Surgical, Medical and Molecular Pathology and Critical Care Medicine, University of Pisa, 56020 Pisa, Italy; elisabetta.carli@for.unipi.it; 3 Dental School, IRCCS San Raffaele Hospital, Vita-Salute San Raffaele University, 20132 Milan, Italy

**Keywords:** elastodontic devices, removable orthodontic appliances, deformation, compression load, mechanical properties, preventive orthodontics, pre-formed appliances

## Abstract

The purpose of the present study was to evaluate the mechanical resistance of elastodontic devices (ED): their maximum compression loads and plastic deformation under loading (percentage). An Instron universal machine (Model 3365, Instron, Industrial Product Group, Grove City, PA, USA) was employed with a 100 N load cell and with Bluehill software for loading analyses. Each device was submitted to a five-cycles test. The following ED were evaluated: A.M.C.O.P. (Micerium, Genova, Italy) in red color, in orange color, and in blue color; HealthyStart (Ortho-Tain, Winnetka, IL, USA), and T4K™ phase 1 (Myofunctional Research Co., Helensvale, Australia). During the five-cycles test, the Ortho-Tain device delivered the greatest compression load (7.56 N), with the lowest percentage of deformation (0.95%). For all devices, a slight plastic deformation of the material was registered, ranging from 0.95% to 1.75%. For the T4K device it was not possible to complete the five-cycles test. For all the analyzed ED, a slight plastic deformation under loading was registered, that in all cases can be considered clinically acceptable. Further studies are needed to test the appliances after clinical usage.

## 1. Introduction

Interceptive orthodontics represents a preventive approach for treating malocclusion at pediatric age [1]. It has been suggested that orthodontic problems in mixed dentition can be corrected with interceptive treatment in 15% of patients and improved in 49% of cases [1]. In the 1980s prefabricated functional appliances were commercially introduced from manufacturers, available under different proprietary names, to be used also with children in the period of primary dentition [2]. These removable appliances are typically made of a soft elastomeric material, and incorporate soft tissue shields around the dentition with the aim of correcting overjet and overbite by combining the features of a functional appliance (forward mandibular posture) with eruption guidance (soft tissue shields) [3]. This approach was recently named “elastodontics”, i.e., a particular type of orthodontic treatment using prefabricated elastodontic devices (ED). The characteristic of these appliances is that they are made with silicone elastomer which has optimal characteristics of resilience, guiding the teeth and the mandible to a correct position. Considering that these appliances are simple in construction and function, easy to use and safe, their use has become more and more diffused [4].

ED produce light and biological elastic forces to correct malocclusions, changing the position of the teeth and potentially affecting growth of the bone bases, by stimulating the perioral muscles [5] and remodeling and relocation of the glenoid fossa. These appliances are used with children with primary dentition as, when muscular interferences or dento-skeletal malocclusions are treated at a very early stage, it seems to improve the stability of relationship between the jaws, and their muscular component [6,7]. Their main clinical applications described in the literature are for increased overjet and overbite, gummy smile, anterior crowding and rotations, open bite, class II malocclusion, and scissor bite [8]. Overall, they are considered to act globally on the stomatognathic system of growing children, by perfectly integrating with the neuromuscular system [9,10]. In addition, elastodontics became popular during the pandemic (2020–2021) as their characteristics are suitable for remote monitoring and tele dentistry [11,12].

Today, orthodontists have access to a wide range of ED.

One of the most popular is the so-called trainer for kids, the T4K™ (Myofunctional Research Co., Helensvale, Australia), a polyurethane appliance, suggested to correct class II malocclusions [13]; furthermore, it is also considered to stimulate transverse development [14].

Another recently developed elastodontic approach is represented by the bio-activator A.M.C.O.P.™ (cranium-occluded-postural multifunctional harmonizers) (Micerium, Genova, Italy). These appliances consist of two flanges, one vestibular and one lingual, which delimit a free central area, allowing an orthopedic effect, while moving without pressure [15,16]. It can be considered one of the most popular in the market, because several of these devices are available for different types of malocclusions, distinguished by different colorations and transversal sizes [17]. Being applied for all types of malocclusions, they allow complete orthodontic treatment until the alignment of teeth, often without any need of refinement with other appliances [18]. Thus, they have become popular among clinicians.

Another popular ED is the HealthyStart™ (Ortho-Tain, Winnetka, IL, USA), specifically suggested for oral breathing children to reduce this muscular dysfunction, while developing the jaw naturally and straightening teeth.

These appliances have been described to be clinically capable of improving skeletal patterns as Class II malocclusions, at an early age in a relatively cost-effective manner, by acting on muscular dysfunction and repositioning the mandible [13]. In the literature, these appliances were investigated in clinical cases of class II malocclusion, and were described as influencing the sagittal plane in class II malocclusion, in a manner comparable to that of other functional devices such as the twin-block [19], but are less effective than other appliances as the activator [20].

Despite the existence of studies of their clinical effectiveness, their mechanical characteristics—the assumption of their clinical effectiveness, with respect to other types of functional appliances—have not been analyzed in the literature as yet [2]. Only one study previously evaluated the tensile strength of elastomer appliances comparing three types of appliance, focusing mostly on their tensile strength after an elongation, but that study did not evaluate deformation under loading [21].

Thus, the purpose of the present study is to evaluate the compression resistance of the most popular ED, through the analysis of maximum compression loads and plastic deformation under loading.

## 2. Materials and Methods

This in-vitro study was aimed at evaluating the maximum compression resistance of some popular EDs, as well as their degree of plastic deformation before breaking. The study was carried out at the Engineering Department of the Leone Company (Firenze, Italy). The following ED were evaluated in this study:

-A.M.C.O.P. (Micerium, Genova, Italy) in red color (Ref. ΦS3–55 mm).
-A.M.C.O.P. in orange color (Ref. SC3–55 mm).
-A.M.C.O.P. in blue color (Ref. S3–55 mm).
-HealthyStart (Ortho-Tain, Winnetka, IL, USA) (Ref. 7–11 PAD).
-T4K phase 1(Myofuncional Research Co., Helensvale, Australia) (Ref. T4K).

**Figure 1:**
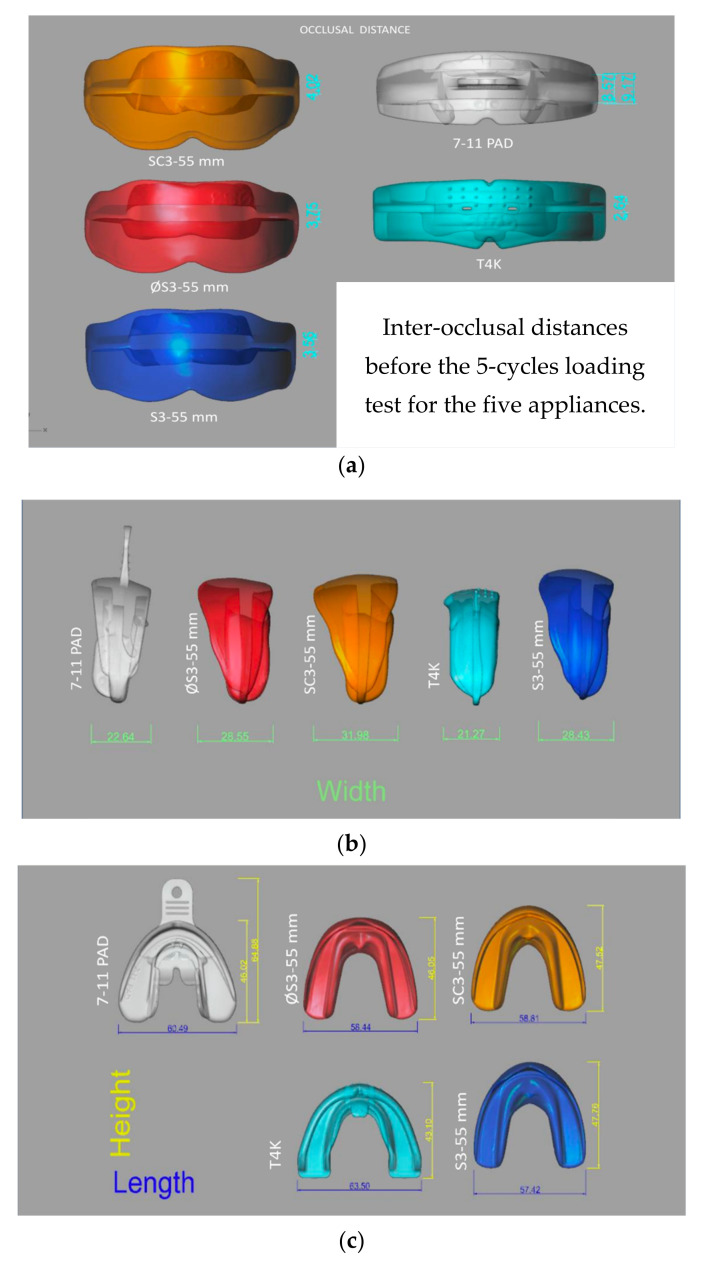
Measurements obtained for the five devices before the 5-cycles test, in millimeters: (**a**) occlusal distance; (**b**) width; (**c**) length and height.

The engineering drawings for every ED are represented in Figure 1, which reports the scanned images of the ED, with dimensions for the occlusal distances (Figure 1a), the width (Figure 1b), the length and the height (Figure 1c), in millimeters.

### 2.1. The Mechanical Tests

A compression test was performed for the mechanical characterization of the devices. An Instron universal traction machine (Model 3365, Instron, Industrial Product Group, Grove City, PA, USA) was employed with a 100 N load cell, with Bluehill software for the analyses.

**Figure 2:**
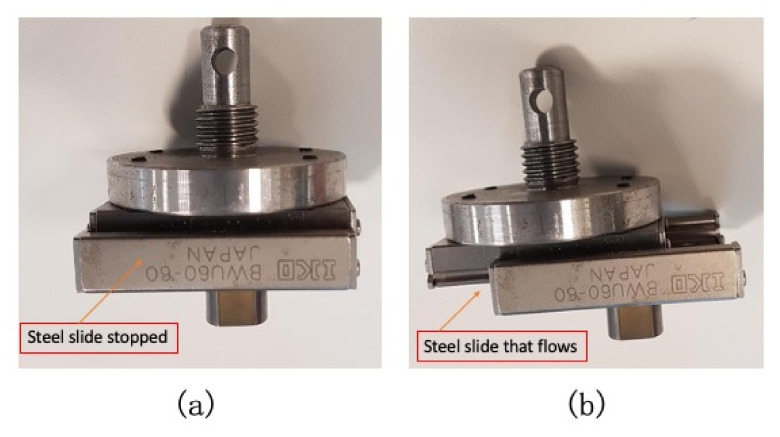
(**a**) The steel slide (BWU60-60, Iko, Japan) that allowed the displacement of the device during the 5-cycles test; (**b**) the steel sliding, allowing the 10 mm displacement of the device at 0.2 mm/s.

Preliminarily, the devices were positioned on a stainless-steel plate in such a way as to have their median line parallel to the crossbar of the traction machine; a synthetic paste was used to hold the device in place and avoid its movement on the transversal plane. For testing, the displacement was applied with a punch on a steel slide (BWU60-60, Iko, Japan) that allowed the force to be applied on the same region of the device during the whole test (it was a 5-cycles test). Thanks to its thread, the punch was installed directly on the crossbar of the traction machine, as represented in Figure 2.

**Figure 3:**
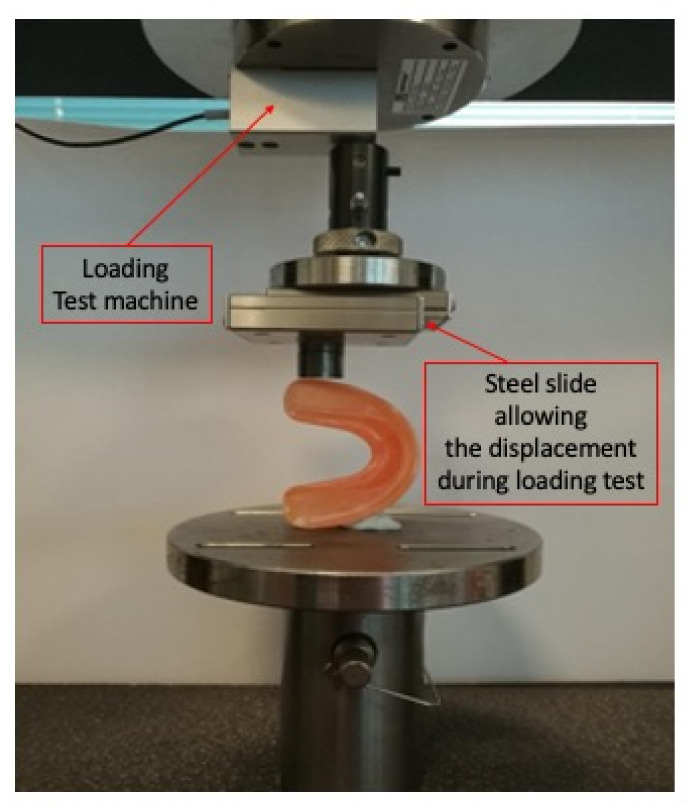
Example of appliance positioning in the mechanical test machine.

A displacement method was developed, as represented in Figure 3: the crossbar dropped down to register 0.1 N; then, a downward displacement of 10 mm, at 0.2 mm/s, was applied during which the machine registered the needed force; a two-seconds pause followed; finally, an upward displacement of 10 mm to 0.2 mm/s was carried out during which the machine registered the force. The same cycle was repeated 5 times.

The variables investigated were the maximum compression load and the plastic deformation under loading. These variables are useful to understand the elastic/plastic behavior of the appliances when subjected to a compressive load and displacement. The variables are described in the following sentences.

### 2.2. The Maximum Compression Loads

Compression is one of the elementary stresses that a body can be subjected to, along with traction, bending, shear and torsion. A body is subject to compression when a system of converging forces acts upon it. In a generic section of a beam subject to compression, the unit stress is calculated via a relation, in which compression force = N/A (in which N is the maximum compression force, and A is the cross-sectional area of the beam, measured in mm^2^). This formula, that was obtained for the calculation of the tensile stress, can be identically applied to compressed bodies. In the present study the maximum loads necessary for compression of each device were calculated as the higher level of force recorded at the maximum stroke during the 5-cycles test for each device. The maximum compression load can be evidenced in a graph where *X*-axis represents the displacement expressed in millimeters, and the *Y*-axis shows the applied load, expressed in N.

### 2.3. The Plastic Deformation under Loading

Plastic deformation of a material occurs when a force is applied to that material in such a way that, when the stress is removed, the material is no longer able to return to its original size, and that force is said to be correlated to a percentage of deformation. Deformation is simply the difference between the original dimensions and the new dimensions that the material acquires [22]. When a material remains with dimensions different from the original when stress is removed, it is said to have been plastically deformed. Plastic deformation is achieved when the applied force is so great that, internally, the atoms or molecules have had to change position to compensate for this force. In the present study, plastic deformation was calculated on the 3D-scans of the devices taken before and after the 5-cycles test. A scanner Maestro 3D MDS 500 (AGE Solutions s.r.l., Pontedera, Pisa, Italy) was used for scanning the ED, after opacifying them by a white spray paint. The pre- and post-test scans were compared to calculate the percentage of plastic deformation, using the following formula:

Deformation (%) = Start dimension (mm) – Dimension after test (mm) × 100 Start dimension (mm). 

The software used for the measurements were Rhinoceros (Materforma, Terni, Italy), and Netfabb (Autodesk, San Rafael, CA, USA) with specific tools.

**Figure 4:**
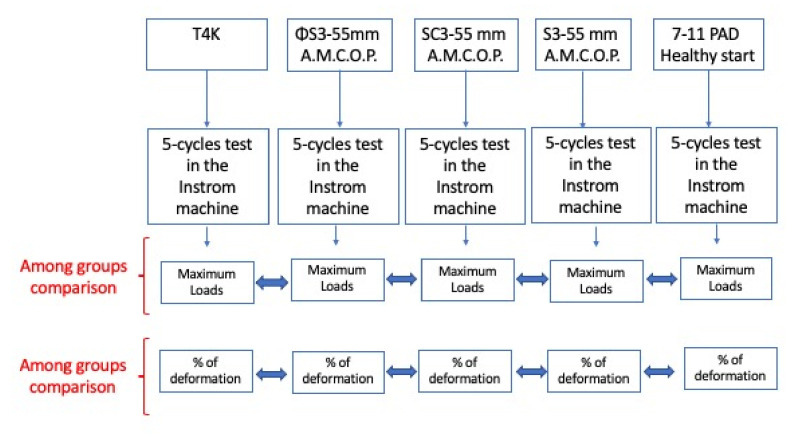
Research experimental workflow.

The research experimental workflow is represented in Figure 4.

## 3. Results

**Figure 5:**
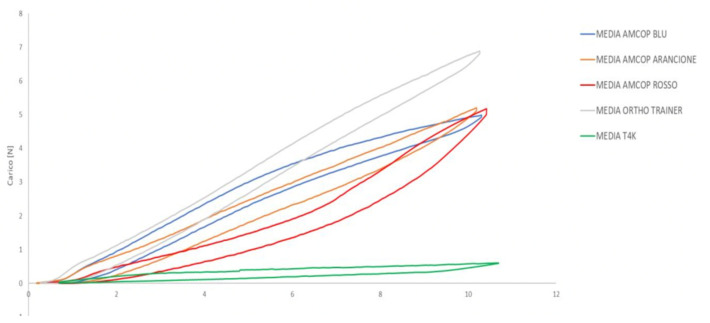
Average compression and release curves of the devices. *X*-axis represents the displacement expressed in millimeters; *Y*-axis shows the applied load, expressed in N.

Figure 5 shows the average compression and release curves of the five cycles for each device.

It can be seen from Figure 5 that it was not possible to complete the five cycles envisaged by the test for the myofunctional research into the T4K device. The test was repeated several times but to keep the device in position during the test it was necessary to introduce metallic elements that would invalidate the test itself. The maximum compression forces for the ED are reported in Table 1. For all devices, a slight plastic deformation of the material as shown in Table 2.

## 4. Discussion

In the present study, EDs were studied to analyze their mechanical characteristics during a compression test with a 100 N load cell and displacement. Maximum compression loads and plastic deformation as a percentage were investigated comparing measurements among the five EDs.

From a clinical point of view, mechanical characteristics of these devices, such as the maximum compression loads and the subsequent plastic deformation, are strategic in terms of their clinical effectiveness in intercepting and treating malocclusion at an early age. When these mechanical characteristics are altered, it leads to a reduction in the clinical effect of the device. Ideally, an ED should not show any deformation under compressive loading during displacement. Obviously, it is expected that a deformation, although minimal, can occur for each device, but the scientific literature does not report standard and/or clinically acceptable data for the percentage of deformation under compressive load for the ED in the market. Thus, in the present study, no standard values were compared to the present ones.

This is because their elastic potential energy, that is the energy stored by their elastic deformation, is the principle on which their mechanism of action is based during their clinical usage. These appliances may be applied in several different clinical situations, and can potentially be useful for moving individual teeth, blocks of teeth, and aiding growth modification as functional conventional appliances [23]. Thus, their clinical effectiveness depends on their elastic potential energy. The elastic potential energy stored by an ED is directly correlated to (1) the amount by which the ED is deformed (in this case, after compression); (2) the amount of force required to plastically deform the ED. These physical properties depend on both the shape and the material used for its manufacturing, as assessed also for the mechanical performance of other devices, such as implants for femoral head against the acetabular cup, that are subjected to a compressive loading during their movements [24].

Therefore, the elastic potential energy can be different for every ED, potentially explaining the clinical results achieved after their usage.

In the present study, for the T4K™ it was not possible to complete the test due to the need to insert metallic elements.

For the other considered EDs, during the five-cycles test, the appliance that brought the greatest compression load was the HealthyStart Ortho-Tain. Regarding plastic deformation, all devices analyzed in this study were subjected to slight plastic deformation during the whole five-cycles test, ranging from 0.95% to 2.55%, with the Ortho-Tain registering the lowest percentage of plastic deformation.

The A.M.C.O.P. device is made of a polymer/elastomer combination, aimed at being elastic and non-deformable. Another characteristic of this appliance lies in the soft consistency of the material, which allows the possibility of performing myofunctional exercises for the oral and lingual musculature while wearing the device during the day. In addition, it is thermoactivated, and extremely adaptable to different arch shapes. At the same time, being soft, possible interferences from flanges can be modified with heat-appropriate instruments; in addition, the patient can also expand the device by soaking it in hot water at about 70 °C for 30 s. Then, to fix it in its new form, it can be soaked in cold water [15]. These characteristics could explain the results obtained in the present study, in which this appliance showed a plastic deformation of about 1.29–1.75%, higher than the Ortho-Tain device, probably due to the thermo-activity of the polymer that requests a plasticity of the material to ensure these clinical characteristics fo4 this device. Therefore, its soft consistence and thermo-activity may explain the higher plastic deformation registered after this mechanical test, compared to the HealthyStart.

Differently, the HealthyStart appliance is made of a vinyl resin whose primary characteristic is resilience. Indeed, it is usually recommended for obtaining minor tooth movements after an orthodontic treatment because of its elastomeric material [25]. In children with primary dentition, it is recommended to be worn during the night and is not projected to allow myofunctional exercises, except for micro-movements. These characteristics are suitable for a more resistant appliance, less deformable under loading, as observed in the present study.

In the previous literature, these appliances have never been compared for these variables, but only in their tensile strength, for which the different materials showed almost the same values: high temperature vulcanizing silicone appliances evidenced similar values compared with urethane and vinyl materials [21].

Regarding deformation under loading, an in-vitro study was performed in the literature [26], but not using appliances, because the dimensional variation of polymer materials was evaluated by using cylinders as material.

In that study, urethane, white rubber, and elastocryl (three materials used to fabricate these types of devices) were compared and the deformation was established during a compression test by compressing cylinders of materials for given periods of time, and then measuring the level of deformation. This study showed that all three materials manifested slight permanent deformation.

Although with a different methodology, the findings agree with that study, and the conclusion is that, from a clinical point of view, that the percentage of deformation was <1.75% for all the analyzed ED can be considered clinically acceptable for their use. The slight difference among the appliances relates to the recommended clinical use.

Regarding the clinical relevance of the present findings, it can be highlighted that mechanical properties and slightly different mechanical characteristics of different EDs can explain their clinically recommended usage. Thus, from the present study it can be stated that the HealthyStart appliance is made of a vinyl resin whose primary characteristic is resilience (high maximum loads, and low deformation). Indeed, it is usually recommended for obtaining minor tooth movements after an orthodontic treatment because of its elastomeric material.

Differently, A.M.C.O.P. is made of a thermo-activable polymer that requires a plasticity of the material and ensures extreme adaptability to different arch shapes, allowing myofunctional exercises during the day, thanks to its soft consistency.

The present study was limited, as the appliances were not preliminary subjected to clinical use or to thermocycling to simulate intra-oral use. Thus, the results could not be the same under the two conditions. The characteristic of elasticity for all the materials could be much lower after clinical usage, or after immersion in the water bath, as assessed for other elastics in orthodontics [27,28,29]. Another limitation is that only a few products available in the market have been evaluated, i.e., the most common, but more thorough research should include more products. Therefore, this study can be considered a pilot.

## 5. Conclusions

ED were studied to analyze their mechanical characteristics during a compression test with a 100 N load cell and displacement. During the five-cycles test, the HealthyStart (Ortho-Tain) showed the lowest percentage of deformation (0.95%) with the highes5 maximum compression load (7.56 N) but, for all devices, only a slight plastic deformation of the appliances was registered, ranging from 0.95% to 1.75%, that in all cases can be considered clinically acceptable. Further studies are needed to test more appliances and evaluate data after clinical usage.

## Figures and Tables

**Table 1:** Maximum compression load (in N) and deformation obtained during the test.

	The Maximum Compression Loads (N)	Maximum Deformation (mm)
A.M.C.O.P. blu color (S3–55 mm)	5.85	10.004
A.M.C.O.P. orange color (SC3–55 mm)	5.33	10.004
A.M.C.O.P. red color (ΦS3–55 mm)	5.81	10.004
HealthyStart (Ortho-Tain) (7–11 PAD)	7.56	10.004
Trainer for Kids (T4K)	0.7	No data *

* It was not possible to complete the 5 cycles of the test for Myofunctional research T4K device.

**Table 2:** Percentage of plastic deformation after 5 cycles.

	Percentage of Deformation (%)
A.M.C.O.P. blu color (S3–55 mm)	1.29
A.M.C.O.P. orange color (SC3–55 mm)	1.41
A.M.C.O.P. red color (ΦS3–55 mm)	1.75
HealthyStart (Ortho-Tain) (7–11 PAD)	0.97
Trainer for Kids (T4K)	2.55

## Data Availability

Data set can be requested to the first author and to the corresponding author.

## References

[1] Graber T.M., Chung D.D., Aoba J.T. (1967). Dentofacial orthopedics versus orthodontics. J. Am. Dent. Assoc..

[2] Wishney M., Darendeliler M.A., Dalci O. (2019). Myofunctional therapy and prefabricated functional appliances: An overview of the history and evidence. Aust. Dent. J..

[3] Migliaccio S., Aprile V., Zicari S., Grenci A. (2014). Eruption guidance appliance: A review. Eur. J. Paediatr. Dent..

[4] Keski-Nisula K., Hernesniemi R., Heiskanen M., Keski-Nisula L., Varrela J. (2008). Orthodontic intervention in the early mixed dentition: A prospective, controlled study on the effects of the eruption guidance appliance. Am. J. Orthod. Dentofac. Orthop..

[5] Tecco S., Epifania E., Festa F. (2008). An electromyographic evaluation of bilateral symmetry of masticatory, neck and trunk muscles activity in patients wearing a positioner. J. Oral Rehabil..

[6] Bergersen E.O. (1984). The eruption guidance myofunctional appliances: How it works, how to use it. Funct. Orthod..

[7] Quinzi V., Nota A., Caggiati E., Saccomanno S., Marzo G., Tecco S. (2020). Short-Term Effects of a Myofunctional Appliance on Atypical Swallowing and Lip Strength: A Prospective Study. J. Clin. Med..

[8] Skomro P. (2000). Aparat ortodontyczny z elastomeru silikonowego w ocenie klinicznej i opinii pacjentów po leczeniu niektórych wad zgryzu [Orthodontic appliance made from silicone elastomer, evaluated clinically and from patient opinions after treatment for malocclusion]. Ann. Acad. Med. Stetin..

[9] Laganà G., Cozza P. (2011). Interceptive therapy with elastodontic appliance: Case report. Ann. Stomatol..

[10] Janson G., Nakamura A., Chiqueto K., Castro R., de Freitas M.R., Henriques J.F.C. (2007). Treatment stability with the eruption guidance appliance. Am. J. Orthod. Dentofac. Orthop..

[11] Montenegro V., Inchingolo A.D., Malcangi G., Limongelli L., Marinelli G., Coloccia G., Laudadio C., Patano A., Inchingolo F., Bordea I.R. (2021). Compliance of children with removable functional appliance with microchip integrated during COVID-19 pandemic: A systematic review. J. Biol. Regul. Homeost. Agents..

[12] Patano A., Cirulli N., Beretta M., Plantamura P., Inchingolo A., Inchingolo A., Bordea I., Malcangi G., Marinelli G., Scarano A. (2021). Education Technology in Orthodontics and Paediatric Dentistry during the COVID-19 Pandemic: A Systematic Review. Int. J. Environ. Res. Public Health.

[13] Ramirez-Yañez G., Sidlauskas A., Junior E., Fluter J. (2007). Dimensional Changes in Dental Arches After Treatment with a Prefabricated Functional Appliance. J. Clin. Pediatr. Dent..

[14] Zhang X., He J.-M., Zheng W.-Y. (2021). Comparison of rapid maxillary expansion and pre-fabricated myofunctional appliance for the management of mouth breathers with Class II malocclusion. Eur. Rev. Med. Pharmacol. Sci..

[15] Fichera G., Martina S., Palazzo G., Musumeci R., Leonardi R., Isola G., Giudice A.L. (2021). New Materials for Orthodontic Interceptive Treatment in Primary to Late Mixed Dentition. A Retrospective Study Using Elastodontic Devices. Materials.

[16] Inchingolo A.D., Patano A., Coloccia G., Ceci S., Inchingolo A.M., Marinelli G., Malcangi G., Montenegro V., Laudadio C., Di Pede C. (2022). The Efficacy of a New AMCOP^®^ Elastodontic Protocol for Orthodontic Interceptive Treatment: A Case Series and Literature Overview. Int. J. Environ. Res. Public Health.

[17] Inchingolo A.D., Ceci S., Patano A., Inchingolo A.M., Montenegro V., Di Pede C., Malcangi G., Marinelli G., Coloccia G., Garibaldi M. (2022). Elastodontic Therapy of Hyperdivergent Class II Patients Using AMCOP^®^ Devices: A Retrospective Study. Appl. Sci..

[18] Cardarelli F., Patano A., Montenegro V., Malcangi G., Coloccia G., Inchingolo A.D., Marinelli G., Laudadio C., Dipalma G., Di Venere D. (2021). Elastodontic therapy un nuovo approccio alla terapia ortodontica funzionale. Il Dent. Mod..

[19] Elhamouly Y., El-Housseiny A.A., Ismail H.A., El Habashy L.M. (2020). Myofunctional Trainer versus Twin Block in Developing Class II Division I Malocclusion: A Randomized Comparative Clinical Trial. Dent. J..

[20] Idris G., Hajeer M.Y., Al-Jundi A. (2018). Soft- and hard-tissue changes following treatment of Class II division 1 malocclusion with Activator versus Trainer: A randomized controlled trial. Eur. J. Orthod..

[21] Warunek S.P., Sorenson S.E., Cunat J.J., Green L.J. (1989). Physical and mechanical properties of elastomers in orthodontic positioners. Am. J. Orthod. Dentofac. Orthop..

[22] Quinn R.S., Yoshikawa D.K. (1985). A reassessment of force magnitude in orthodontics. Am. J. Orthod..

[23] Tecco S., Farronato G., Salini V., Di Meo S., Filippi M.R., Festa F., D’Attilio M. (2005). Evaluation of Cervical Spine Posture After Functional Therapy with FR-2: A Longitudinal Study. CRANIO®.

[24] Jamari J., Ammarullah M.I., Santoso G., Sugiharto S., Supriyono T., Prakoso A.T., Basri H., van der Heide E. (2022). Computational Contact Pressure Prediction of CoCrMo, SS 316L and Ti6Al4V Femoral Head against UHMWPE Acetabular Cup under Gait Cycle. J. Funct. Biomater..

[25] Bergersen E.O., Stevens-Green B., Rosellini E. (2022). Efficacy of Preformed Sleep and Habit Appliances to Modify Symptoms of Sleep-Disordered Breathing and Oral Habits in Children with Focus on Resolution of Mouth Breathing. Compend. Contin. Educ. Dent..

[26] Collett A.R., Cook W.D., West V.C. (1994). Mechanical properties of some polymer materials used for tooth positioners. Aust. Dent. J..

[27] Wong A.K. (1976). Orthodontic elastic materials. Angle Orthod..

[28] Mummolo S., Tieri M., Nota A., Caruso S., Darvizeh A., Albani F., Gatto R., Marzo G., Marchetti E., Quinzi V. (2019). Salivary concentrations of Streptococcus mutans and Lactobacilli during an orthodontic treatment. An observational study comparing fixed and removable orthodontic appliances. Clin. Exp. Dent. Res..

[29] Di Venere D., Pettini F., Nardi G.M., Laforgia A., Stefanachi G., Notaro V., Rapone B., Grassi F.R., Corsalini M. (2017). Correlation between parodontal indexes and orthodontic retainers: Prospective study in a group of 16 patients. Oral Implant..

